# Outcomes of Diffuse Large B-Cell Non-Hodgkin’s Lymphoma After Gemcitabine-Based Second Salvage Chemotherapy: A Single-Center Study

**DOI:** 10.7759/cureus.19699

**Published:** 2021-11-18

**Authors:** Mussadique Ali Jhatial, Manzoor Khan, Saif ur Rab, Naila Shaikh, Chandumal Loohana, Syed W Imam Bokhari

**Affiliations:** 1 Medical Oncology, Shaukat Khanum Memorial Cancer Hospital and Research Centre, Lahore, PAK; 2 Nuclear Medicine, Institution of Nuclear Medicine and Oncology, Lahore, PAK; 3 Medical Oncology, Sindh Institute of Urology and Transplantation, Karachi, PAK

**Keywords:** outcomes, gemcitabine, second salvage chemotherapy, relapsed and refractory dlbcl, non hodgkin's lymphoma

## Abstract

Background

Diffuse large B-cell lymphoma (DLBCL) is the most common subtype of non-Hodgkin’s lymphoma with a five-year survival of 60%-70% with chemoimmunotherapy consisting of the R-CHOP combination (rituximab, cyclophosphamide, vincristine, doxorubicin, and prednisone), with a relapse/refractory rate of 20-50%. Salvage therapy with HDT-ASCT is the treatment of choice for patients with relapsed/refractory disease with a success rate of 50%-60%. Patients who do not respond to the first salvage regimen or who relapsed after the first salvage regimen, with or without high-dose chemotherapy (HDT)-autologous stem cell transplantation (ASCT), have poor overall responses and survival and should be offered novel therapies. The objective of our study was to evaluate responses to second salvage, gemcitabine-based therapy with or without HDT-ASCT in a resource-limited setting.

Materials and methods

This was a retrospective study, including 55 patients aged >18 years, diagnosed with DLBCL and having received gemcitabine-based second salvage chemotherapy.

Results

The median age was 34 years, only one patient achieved progression-free survival (PFS) of >12 months with ORR of 27% to two cycles of gemcitabine-based combination, two years PFS and OS of 9.6% and 34%, respectively, and a median PFS and OS of four months and 13 months, respectively.

Conclusion

DLBCL patients, refractory to first-line and first salvage chemotherapy, should be considered for novel therapies or opt for palliative care rather than second salvage chemotherapy and HDT-ASCT, which results in poor overall response and significant toxicities.

## Introduction

Non-Hodgkin’s lymphoma is ranked the sixth most common malignancy in Pakistan as per Globocan 2018 data with 5876 patients registered in 2018 amounting to 3.4% of the total cancer patient population [1]. Diffuse large B-cell lymphoma (DLBCL) is the most common subtype of non-Hodgkin lymphoma (NHL) [2] with a five-year survival rate of around 60%-70% [3]; around 50% of patients become refractory to treatment or relapse subsequently [4]. Before the advent of rituximab, combination therapy with cyclophosphamide, vincristine, doxorubicin, and prednisone (CHOP) was considered the standard first-line treatment [5]. The addition of rituximab, an anti-CD-20 monoclonal antibody, to the standard CHOP-like combination in elderly patients yielded significant improvement in event-free survival (EFS) and overall survival (OS) with complete responses of 76% and OS of 70% at two years in the rituximab group as compared to 63% and 57%, respectively, in those who did not receive rituximab [6-7]. These results were further confirmed by a trial conducted in the younger patient population with a six-year EFS of 74% with rituximab as compared to 56% without rituximab. A further subgroup analysis showed that better EFS was determined by a favorable disease profile with an international prognostic index (IPI) score of 'zero’ as compared to one or more and non-bulky as compared to bulky disease (six-year EFS 84% and 71%, respectively) [8]. Similarly, response to treatment and prognosis is determined by the revised international prognostic index (R-IPI) score based on age, stage, Eastern Cooperative Oncology Group Performance Scale (ECOG-PS), lactate dehydrogenase (LDH), and the number of extranodal sites, and those with an R-IPI of ‘Zero’ have a very good prognosis with a four-year progression-free survival (PFS) and OS of 94% each as compared to PFS and OS of 53% and 55%, respectively, for an R-IPI score of 3 and above [9]. Approximately, 20%-50% patients remain refractory (10%-15%) to or relapse (30-40%) after complete response post-treatment with the rituximab and CHOP combination [2,10-11].

The strategy for transplant-eligible patients with refractory or relapsed [12] DLBCL is treatment with salvage chemotherapy followed by high-dose therapy and autologous stem cell transplantation (HDT-ASCT) [13-16]. If not treated further with salvage therapy with or without a transplant, these relapsed and refractory patients have an average life expectancy of fewer than six months [12] and treatment with salvage chemotherapy alone has poor overall outcomes [15-18]. However, around 40%-50% of these relapsed or refractory DLBCL patients do not respond with either complete remission (CR) or partial remission (PR) to first salvage chemotherapy and fail to proceed with HDT-ASCT with a poor prognosis and a median overall survival of six months. Out of the remainder, 50%-60% who do respond to first salvage chemotherapy and proceed with HDT-ASCT, 30%-40% suffer from a relapse post-HDT-ASCT [19] and have a median survival of around three months [20]. Hence, around 75% of patients with relapsed or refractory DLBCL end up being considered for second salvage chemotherapy. It has been shown that these relapsed patients have a poor prognosis if their secondary IPI score at the time of relapse is two or more or if they have relapsed within 12 months of HDT-ASCT [21]. Overall, the utility of second salvage chemotherapy for those who do not respond to first-line treatment and first salvage chemotherapy is in the order of 14%-32%, and as a result, there is little chance of cure for most of the patients who have refractory DLBCL [22]. In particular, patients with refractory DLBCL have a CR rate of only 7% and a dismal median OS of six months. Subsequent lines of chemotherapies, in turn, also have significantly more toxicities requiring more supportive care, hospital visits and stay, and hence an alternative better option may be either to go for novel therapeutic agents or chimeric antigen receptor T cells (CAR-T cells) if available or palliative care [23].

The treatment approach, at a number of centers including ours, of relapsed or refractory DLBCL patients not responding to first salvage or relapsing after HDT-ASCT has been to offer Gemcitabine-based second salvage chemotherapy. This is partly driven by resource constraints and the non-affordability or non-availability of novel therapeutic agents. We have conducted a retrospective study to assess outcomes of gemcitabine-based second salvage chemotherapy at our center. Our primary objective was to assess efficacy and outcomes and secondary objectives were to identify any significant factors or sub-groups with significantly different outcomes.

This article was previously presented as a meeting abstract at the JSMO2021 Virtual Congress on February 18-21, 2021.

## Materials and methods

Objectives

The primary aim of this study was to assess response rates and outcomes in terms of PFS and OS in relapsed or refractory DLBCL patients treated with gemcitabine-based second salvage therapy. The secondary aim was to assess the effect of various factors on PFS and OS; like the stage at diagnosis, baseline R-IPI, response to first-line chemotherapy, duration of response to the first-line regimen, prior exposure to rituximab, and stage at second relapse.

Methodology

This was a retrospective study that included patients with DLBCL, registered at our institute during the period from June 1994 to June 2019, who relapsed or were refractory to first-line and first salvage chemotherapy with or without consolidation with autologous stem cell transplant and went on to receive gemcitabine-based second salvage chemotherapy.

Patient selection

Patients aged 18 and above, diagnosed with DLBCL were included. Low-grade lymphomas and Burkitt’s lymphoma were excluded from the study. Patients lacking minimal essential data as described below were excluded.

Data collection

Data were collected from the hospital information system (HIS) with a keyword search for non-Hodgkin’s lymphoma and gemcitabine. The data were further scrutinized for those who received second salvage gemcitabine-based chemotherapy. Data collected included age, gender, comorbid medical conditions, ECOG-PS, LDH, IPI, and stage of disease at the time of the start of first-line chemotherapy, upfront treatment regimen, anti-CD20 treatment, first salvage, autologous HCT, responses and their duration, second salvage chemotherapy, number of cycles received, response to second salvage with duration, relapse, or progression, and date of death. Minimal essential data required included treatments used in the first line, first salvage, and second salvage, response to second salvage, date of relapse post-second salvage, last follow-up, and date and cause of death.

Data analysis

The data were tabulated and analyzed using SPSS for Microsoft Windows, version 21.0 (IBM Corp., Armonk, NY). Age was described as median and range, frequency was tabulated as percentage for gender, ECOG-PS, LDH, stage, R-IPI, chemotherapy regimen, rituximab exposure, treatment response, as well as the duration of response at baseline, at first relapse as well at second relapse. Treatment responses were documented as per standard lymphoma response criteria as complete response (CR), partial response (PR), stable disease (SD), or progressive disease (PD). OS and PFS were calculated from second salvage gemcitabine-based chemotherapy while responses are presented as percentages. Kaplan Meier curves are used to compare survivals over time for the overall population. A further subgroup analysis was carried out with stage at diagnosis; early versus advanced stage, primary R-IPI (at diagnosis), response to first-line chemotherapy, duration of response to the first-line regimen, prior exposure to rituximab, and stage at second relapse, and p values <0.05 were considered statistically significant.

Patients were included in the study after obtaining an exemption from the institutional review board of Shaukat Khanum Memorial Trust (EX-09-03-18-01-A1).

## Results

Baseline characteristics

A total of 124 patients were identified using keyword search with DLBCL and gemcitabine. Data were further scrutinized to obtain 52 patients with a diagnosis of DLBCL and having received second salvage gemcitabine-based chemotherapy. The rest of the patients were excluded because they received gemcitabine-based chemotherapy in either first-line, first salvage, or third salvage settings.

Baseline demographics and clinical characteristics of our patient population are tabulated (Table 1).

**Table 1:** Baseline patient demographics and clinical characteristics ECOG-PS: Eastern Cooperative Oncology Group - Performance Status; LDH: Lactate Dehydrogenase; R-IPI: Revised International Prognostic Index; CHOP: Cyclophosphamide, Hydroxyrubicin (Doxorubicin), Oncovin (Vincristine), Prednisolone; HCVAD: Hyperfractionated Cyclophosphamide, Vincristine, Doxorubicin, Dexamethasone; IDARAM: Idarubicin, Dexamethasone, Cytarabine, and Methotrexate; DA-EPOCH: Dose Adjusted - Etoposide, Prednisolone, Oncovin, Cyclophosphamide, Hydroxyrubicin; CR: Complete Remission; PR: Partial Remission; SD: Stable Disease; PD: Progressive Disease

Age (Range)	18-60 years
Median age	34 years
Gender: n=52 (%)	
Male	38 (73.1%)
Female	14 (26.9%)
Male : Female ratio	2.7
ECOG-PS	
ECOG-PS: 0-1	34 (65%)
ECOG-PS: >2	18 (35%)
Stage; early VS advanced	
Early stage	08 (15%)
Advanced stage	44 (85%)
LDH; normal VS high	
Normal	40 (77%)
High	12 (23%)
R-IPI at baseline	
R-IPI 0	None
R-IPI 1-2	30 (58%)
R-IPI > 3	22 (42%)
First line regimen	
CHOP	49 (94%)
HCVAD	01 (02%)
IDARAM	01 (02%)
DA-EPOCH	01 (02%)
Rituximab in 1^st^ line regimen	
Rituximab	13 (25%)
No rituximab	39 (75%)
EOT response to 1^st^ line regimen	
CR	17 (33%)
SD	09 (17%)
PD	26 (50%)
DOR to 1^st^ line regimen (n=17)	
< 6 months	08/17 (47.1%)
6-12 months	05/17 (29.4%)
> 12 months	04/17 (23.5%)

The age range was 18-60 years, with a median age of 34 years. Out of 52, 38 (73%) were male and 14 (27%) were female. Only eight patients (15%) had stage I-II disease, the rest all had stage III-IV disease (n= 44, 85%). Thirty-four (65%) patients had ECOG-PS of up to one and LDH was high in 40 (75%) out of 52 patients. Thirty out of 52 patients (58%) patients had an R-IPI of one to two and the rest had an R-IPI of three and above. None of the patients were identified with an R-IPI of zero.

First-line regimen

Out of 52, 49 (94%) received CHOP chemotherapy, with upfront rituximab in 13 (25%) patients only. In this cohort, the overall response to first-line CHOP was 33% (n=17); all were in complete remission at the end of treatment. Thirty-five patients of this cohort (67.3%) were refractory, eight patients (15.3%) relapsed within less than six months, five (9.6%) within six to 12 months, and four (7.7%) relapsed after >12 months.

First salvage regimen

Twelve out of 52 patients relapsed as early-stage disease (23%) and the rest as advanced-stage disease (77%). Secondary IPI was one to two for 58% (n=30) and three and above for the rest of 42% (n=22). All 52 patients received one to six cycles of first salvage chemotherapy, with ICE (ifosfamide, cisplatin, etoposide) being the most commonly offered regimen (n=37, 71%) followed by DHAP (dexamethasone, high-dose Ara-C, cisplatin) (n=12, 23%). In this cohort, overall 28 patients (56%) responded with a partial metabolic response and one (2%) with a complete metabolic response. Out of these 29 responders, only one patient received ASCT and the rest continued and completed six cycles of first salvage. Eighteen of the 29 responders could not have ASCT due to social/affordability reasons, four refused and six failed harvesting. Seven out of these 29 responders went on to achieve CR, six with six cycles of ICE, one with HDT-ASCT. Three out of these seven patients received rituximab in addition to ICE. Hence, 23 patients (44.2%) were refractory to first salvage, 26 patients (50%) relapsed within six months, two (3.8%) relapsed between six and 12 months, and one (1.9%) relapsed after > 12 months. Clinical characteristics at first relapse are summarized in Table 2.

**Table 2:** Clinical characteristics at first relapse R-IPI: Revised International Prognostic Index; ICE: Ifosfamide, Cisplatin, Etoposide; DHAP: Dexamethasone, High-Dose Ara-C, Cisplatin; cMR: Complete Metabolic Remission; pMR: Partial Metabolic Remission; SD: Stable Disease, PD: Progressive Disease

Stage at 1^st^ relapse	
Early stage (I&II)	12 (23%)
Advanced stage (III&IV)	40 (77%)
R-IPI 2	
R-IPI 0	None
R-IPI 1-2	30 (58%)
R-IPI > 3	22 (42%)
First salvage regimen	
ICE	37 (71%)
DHAP	12 (23%)
Other	03 (06%)
Rituximab in 1^st^ salvage	
Yes	11 (21%)
No	41 (79%)
Response to 2 cycles of 1^st^ salvage	
cMR	01 (02%)
pMR	28 (54%)
SD/PD	23 (44%)
ASCT consolidation (n=29)	
ASCT received	01 (03%)
ASCT refused	04 (14%
Failed stem cell harvest	06 (21%)
Not offered ASCT	18 (62%)
EOT response to 1^st^ salvage	
cMR	06 (21%)
SD	07 (24%)
PD	16 (55%)
DOR to 1^st^ salvage (n=29)	
< 6 months	24 (83%)
6-12 months	04 (14%)
> 12 months	01 (03%)

Responses to second salvage chemotherapy

All 52 patients received gemcitabine-based second salvage chemotherapy, with 1000 mg/m^2^ of gemcitabine on D-1 and D-8. Most (73%, n=38) patients received gemcitabine (1000 mg/m^2^, D-1 & D-8) in combination with dexamethasone 40 mg/day from D-1 to 4 and cisplatin 75 mg/m^2^, 11.53% (n=6) received gemcitabine (1000 mg/m^2^, D-1 & D-8) in combination with carboplatin (11.53%) or vinorelbine (11.53%) plus dexamethasone each, and 3.8% (n=2) received gemcitabine and (1000 mg/m^2^, D-1 & D-8) dexamethasone only. Ten patients (19%) had early-stage and the rest (81%) had advanced-stage disease. Fourteen patients (27%) responded to two cycles of GDP with only one achieving CR. The rest of the 38 patients (73%) proved to be refractory, with five (9.6%) having stable disease and the rest, progressive disease. Two out of the 14 responders to second salvage (%) went on to receive HDT-ASCT. 

Outcomes

Primary outcomes for this study were progression-free survival (PFS) and overall survival (OS). PFS was defined as the time from the start of second salvage chemotherapy to disease progression, change of therapy if no documented disease progression, or last follow-up with the patient in remission. OS was defined as the time from the start of the second salvage chemotherapy to death from any cause or last follow-up.

Progression-Free Survival

**Figure 1:**
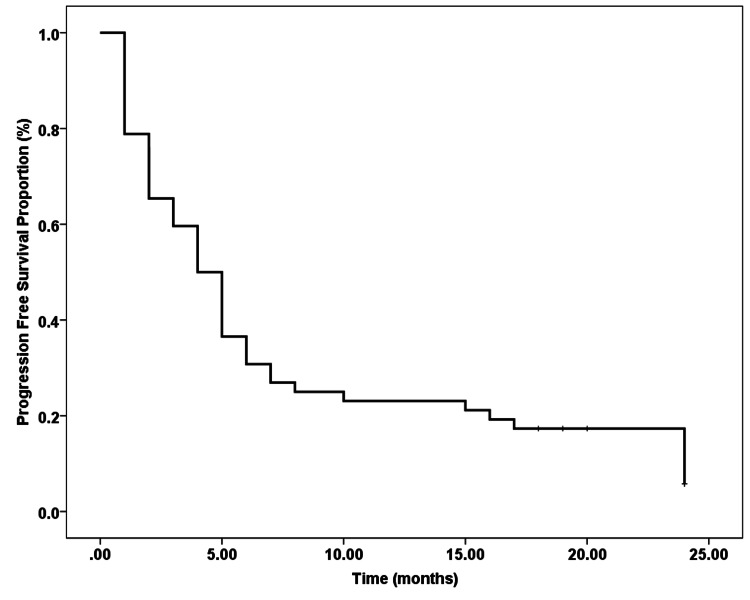
Progression-free survival in months from the commencement of second salvage chemotherapy with or without autologous transplant

PFS of > 12 months was achieved in only five patients (9.6%), two of these with HDT-ASCT and three with four to six cycles of GDP. Two-year PFS was 9.6% with a median PFS of four months (Figure 1).

Overall Survival

**Figure 2:**
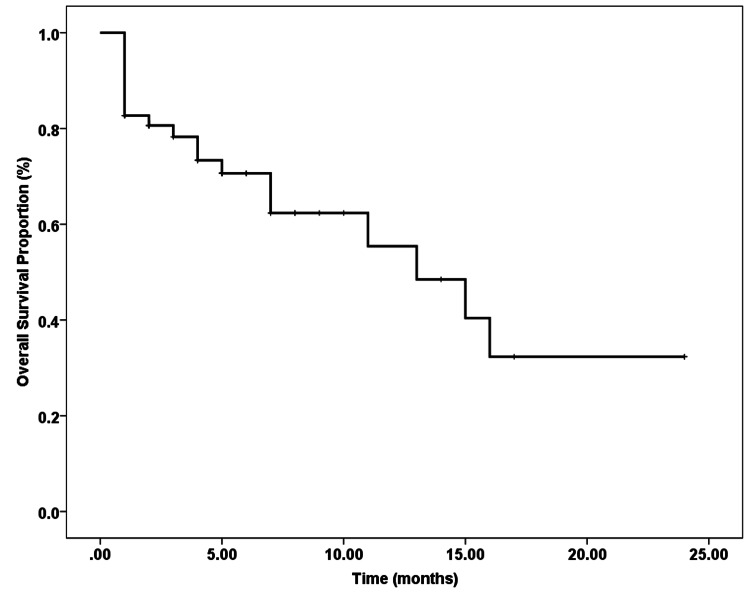
Overall survival in months from commencement of second salvage chemotherapy with or without autologous transplant

Two-year OS was 34% with median overall survival of 13 months (Figure 2).

Univariate Analysis

Univariate analysis of this cohort was carried out with respect to the stage at diagnosis, early versus advanced stage, primary R-IPI (at diagnosis), response to first-line chemotherapy, duration of response to the first-line regimen, prior exposure to Rituximab, and stage at second relapse. None of the subsets showed any significant effect on outcomes with non-significant p-values (Table 3).

**Table 3:** Univariate analysis: overall and progression-free survival by patient subgroups R-IPI: Revised International Prognostic Index; CR: Complete Remission

Subgroups	OS (p-value)	PFS (p-value)
Early stage vs advanced stage at diagnosis	0.848	0.664
R-IPI at diagnosis; 1-2 and >3	0.357	0.374
Response to 1^st^ line regimen; CR vs no CR	0.622	0.386
Duration of response to 1^st^ line regimen; < 6-12 vs > 12 months	0.811	0.273
Exposure to rituximab, none vs received in 1^st^ line or 1^st^ salvage	0.513	0.340
Stage at second relapse; early vs advanced stage	0.516	0.430

## Discussion

The first-line standard of care for DLBCL is chemo-immunotherapy with R-CHOP. In case of relapse or refractoriness, salvage chemotherapy followed by HDT/ASCT is the standard of care. Refractoriness to first salvage or relapse after HDT-ASCT has remained an area of unmet need until recently where novel therapies including antibody-drug conjugates, bi-specific T-cell engagers (BiTE) antibodies, checkpoint inhibitors, and CAR-T cell therapy have provided hope. However, these novel therapies are expensive and not readily available, particularly in resource constraint regions. Hence, second salvage chemotherapy, particularly gemcitabine-based regimens have and are still being utilized in this patient population despite a paucity of data in favor of its efficacy. Our study was conducted to identify whether second salvage chemotherapy provided any long-term progression-free or overall survival. A number of factors have been previously identified to significantly affect responses and outcomes to further treatment, including time to relapse from initial treatment, use of and response to upfront rituximab, and secondary age-adjusted IPI [24]. Refractoriness to, or early relapse after, CHOP chemotherapy is considered an indicator of poor response to second-line chemotherapy regimens [25]. Responses to three cycles of salvage chemotherapy, R-ICE, or R-DHAP have been documented to be around 63% in one study [26] and in patients who do not undergo high-dose chemotherapy and autologous transplant, the response to three to four additional cycles of chemotherapy is of the order of 40%-45% with overall survival of only 32% [15]. Another study with a small number of relatively older age patients documents overall response to one to six cycles of salvage R-ICE of 36% with a three-year PFS and OS of 32% and 35% [27], and a study from China reports an objective response rate of 49% to two to three cycles of the first salvage regimen, including ICE or DHAP [28]. Our study also analyzed different factors that may identify groups of patients where second salvage may still have a role in prolonging progression-free and overall survival.

Our study shows quite poor progression-free and overall survival with gemcitabine-based second salvage chemotherapy. The median PFS and median OS of our patient population were four months (Figure 1) and 13 months (Figure 2), respectively, which is similar to that documented in contemporary literature (median PFS 2.1 months and median OS of 11.4 months) [29]. These poor outcomes, we think, are partly due to the lack of, or the late introduction of, rituximab in the treatment pathway, i.e. during first or second salvage, and likely to be due to the lack of earlier/timely utilization of HDT-ASCT during salvage treatment. Our study also confirms that patients refractory to first-line and first salvage therapy have the worst PFS as well as OS and, hence, treating these patients with gemcitabine-based second salvage is futile. However, those patients who were not refractory to the first line and first salvage, who could not receive rituximab and/or HDT-ASCT in their earlier treatment regimens, still had a trend towards better PFS and OS.

Apart from being a retrospective study, the major limitation of our patient population is that most of the patients could not get rituximab upfront and most of them could not be offered high-dose chemotherapy and ASCT, both because of non-affordability. Non-affordability is a major issue in the developing world and a big limitation for proper treatment. Therefore, the results cannot be generalized to the population in general; however, this study can guide physicians to timely palliate the patients if rituximab, ASCT, or novel therapies are not available or not affordable.

## Conclusions

Primary refractory patients to first-line and first salvage chemotherapy perhaps should be considered for novel therapies, such as antibody-drug conjugates, BiTE antibodies, or CAR-T cell therapy, if available, or they should go for palliative care. Pursuing gemcitabine-based second salvage in these patients will only give more toxicities and waste resources with increased hospital visits and stay; instead, the same resources perhaps should be utilized in the usage of rituximab upfront and HDT-HSCT with first salvage. On the other hand, until novel therapies, such as antibody-drug conjugates, BiTE antibodies, or CAR-T cell therapy are available or affordable in resource-constrained regions, our study shows that it is reasonable to consider gemcitabine-based second salvage in patients who are not primary refractory and/or could not receive HDT-ASCT after first salvage or have not received rituximab in earlier therapies.
